# Krüppel-like Factor 4-Deficient Cells Are Sensitive to Etoposide-Induced DNA Damage

**DOI:** 10.3390/cimb47040217

**Published:** 2025-03-21

**Authors:** Maxwell H. Rubinstein, Aidan Conroy, Elisabeth L. Pezzuto, Hadeel Al Qoronz, Patrick Wertimer, Engda G. Hagos

**Affiliations:** 1 Department of Biology, Colgate University, Hamilton, NY 13346, USA; mrubinstein@colgate.edu (M.H.R.); aac4008@med.cornell.edu (A.C.); elisabeth.pezzuto@kitz-heidelberg.de (E.L.P.); halqoronz@colgate.edu (H.A.Q.); pwertimer@rockefeller.edu (P.W.); 2 Jill Roberts Institute for Research in Inflammatory Bowel Disease, Weill Cornell Medicine, New York, NY 10021, USA; 3 KiTZ—Hopp Children’s Cancer Center Heidelberg, 69120 Heidelberg, Germany; 4 Laboratory of Molecular Biology, Rockefeller University, New York, NY 10065, USA

**Keywords:** Krüppel-like factor 4, DNA damage, genomic instability, cell cycle, DNA damage response, DNA repair

## Abstract

Krüppel-like factor 4 (KLF4) is a highly conserved zinc-finger transcription factor involved in cellular processes such as development, differentiation, and cell cycle regulation. Previous studies show that mouse embryonic fibroblasts (MEFs) null for *Klf4* exhibit increased genomic instability. While KLF4 is regarded as a tumor suppressor in many human cancers, its role in DNA repair mechanisms remains unknown. In this study, cultured MEFs wild type (+/+) and null (−/−) for *Klf4* and human carcinoma colorectal (RKO) cells were studied as a model for human colorectal cancer. Etoposide, a chemotherapeutic topoisomerase II poison, was employed to investigate KLF4’s role in DNA damage repair. Following etoposide treatment, immunostaining and Western blotting revealed cells expressing Klf4 exhibited lower levels of γ-H2AX, a biomarker for DNA damage, compared to cells without *Klf4*. Moreover, after DNA damage, cells expressing *Klf4* exhibited increased levels of BRCA1 and Rad51, known tumor suppressor genes. Finally, genes involved in DNA damage response (DDR), ATR, and Chk1 were upregulated in cells containing functional KLF4, offering a possible mechanism for KLF4’s role in mediating DDR. Our results indicate that KLF4 plays a crucial role in maintaining genetic stability by enhancing cell DDR, supporting previous findings that KLF4 functions as a tumor suppressor.

## 1. Introduction

Krüppel-like factor 4 (KLF4) is a zinc-finger transcriptional factor found primarily in epithelial cells of the gastrointestinal tract, and is involved in many cellular processes, such as autophagy, mitophagy, proliferation, and apoptosis [1,2,3,4]. KLF4 is of interest due to its tumor suppressor functionality in various types of cancer cells, most notably, in colorectal cancer (CRC) cell lines [5,6,7,8,9]. Furthermore, KLF4 is downregulated in colorectal cancer, promoting uncontrolled cell proliferation and metastasis, which are hallmarks of cancerous cell types [7]. Specifically, KLF4 has been reported to be activated by p53, which activates the transcription of cyclin-dependent kinase inhibitor p21, to inhibit proliferation and arrest cells at either the G1 or G2 phase of the cell cycle [10,11,12].

The role of genetic instability is of interest since it is the “hallmark” of cancer and places a large burden on human health. We previously showed that mouse embryonic fibroblasts (MEFs) null for *Klf4* (*Klf4^−/−^*) displayed higher genetic instability, including chromosomal aberrations and breakages, compared to MEFs wild type for *Klf4* (*Klf4^+/+^*), indicating Klf4’s role in maintaining genetic stability in primary cell lines [13]. In response to DNA double-strand breaks (DSBs), the histone variant Ser-139 residue is phosphorylated to form γ-H2AX, a known biomarker for DNA damage [14,15]. Further, we showed γ-H2AX levels were elevated in *Klf4*^−/−^ MEFs compared to *Klf4*^+/+^ MEFs, indicating higher levels of DNA damage in the *Klf4*-deficient cells at the basal level and providing further evidence for KLF4’s role in maintaining genetic stability [13].

Chk1 is a serine/threonine kinase that serves as a major effector in the DNA damage response (DDR) pathway, particularly in the activation of cell cycle checkpoints in response to DNA damage [16,17]. Previous studies demonstrated that the ATM-Chk2 pathway signals for early induction of apoptosis, whereas ATR-Chk1 signals for cell cycle arrest upon DNA damage [18,19,20]. The ATR-Chk1 DDR involves cell arrest and stabilization of replication forks to reduce and monitor damage induced at replication sites [21].

In this study, we employed etoposide, a well-known chemotherapeutic agent that poisons topoisomerase II and causes DNA damage [22]. The lesions created by etoposide are known to primarily cause double-strand DNA breaks and activate cell checkpoints by inhibiting the ATR pathway [23,24]. Further, etoposide acts by redistributing replicative proteins during the S phase of the cell cycle [25]. Previous studies have shown that Chk1, a DNA repair protein kinase upregulated by ATR, can decrease the lethality of etoposide by halting the cell cycle at the G2/M boundary and encouraging DNA repair [16]. Following etoposide-induced DNA damage, we demonstrate that cells expressing KLF4 exhibit lower levels of damage relative to cells lacking functional KLF4. Further, our results suggest that in the presence of DNA damage, KLF4 might play a role in promoting BRCA1 foci to specific sites of the damage to arrest cells and encourage repair. However, in RKO cells allowed to recover post DNA damage, there was a slight increase in BRCA1 levels when KLF4 was expressed in comparison to BRCA1 levels in cells not expressing KLF4. Moreover, we found elevated expression levels of cell cycle regulator proteins such as ATR and Chk1 in cells expressing functional KLF4 but observed no notable difference in the expression of ATM. Taken together, our results strongly suggest that KLF4 plays a crucial role in maintaining genetic stability by enhancing cellular DNA repair mechanisms.

## 2. Materials and Methods

### 2.1. Cell Culture and Drug Treatment

The human colorectal cancer RKO-EcR-KLF4 (RKO) cell line was previously transfected with a plasmid to express KLF4 and GFP when treated with Ponasterone-A (Pon-A) [8]. RKO and mouse embryonic fibroblast (MEF) cells were maintained in Dulbecco’s Modified Eagle’s Medium (DMEM) supplemented with 10% fetal bovine serum (FBS) and 1% penicillin–streptomycin (P/S). Cells were incubated at 37 °C in an atmosphere containing 5% CO_2_. At 24 h after plating in 6-well plates, RKO cells were treated with 5–10 μM Pon-A for 72 h to selectively induce KLF4 expression. Pon-A is an analog of the insect hormone ecdysone and is commonly used to induce the expression of specific genes of interest in mammalian cells [26]. KLF4 expression in RKO cells was confirmed by GFP fluorescence. To induce DNA damage, 60 μM of etoposide (ETOP) was added to the plates, and cells were treated for 5 h. A recovery group was established by treating the cells with etoposide for 5 h, washed with phosphate-buffered saline (PBS) placed in fresh DMEM, and allowed to recover for 24 h (ETOP-REC). Control cells were treated with an equivalent amount of DMSO. Cell morphology was assessed using an Olympus U-RFL-T using brightfield and analyzed further with the Infinity 3 software.

### 2.2. Plasmid Transfection

To overexpress *Klf4*, MEFs were transiently transfected with 5 μg DNA plasmids containing *Klf4*-GFP or GFP as a control in a 6-well plate. Lipofectamine 3000 reagent (Life Technologies, Grand Island, NY, USA) was used following the manufacturer’s protocol. Cells were photographed and then collected for Western blot at various time points. Transfection efficiency was examined under an Olympus IX51 microscope monitoring GFP fluorescence 24 h post-transfection.

### 2.3. Western Blot Analysis

Protein extraction and Western blot analyses were performed using standard techniques as previously described [13]. Nitrocellulose membranes were briefly blocked with 5% BSA in TTBS and treated with 1:1000 primary antibodies against KLF4, γ-H2AX, BRCA1, Chk1, Chk2, and actin overnight at 4 °C on a rocker platform. The blots were incubated with 1:2000 dilution anti-rabbit HRP conjugated secondary antibodies for one hour at room temperature. After incubation and washing, visualization solution (1:1 peroxide/luminol solution) was pipetted at the expected molecular weights, and protein expression was visualized and quantified using BioRad image lab (Bio-Rad, Hercules, CA, USA) and quantified relative to actin levels [1].

### 2.4. Immunostaining

RKO cells (60–70% confluency) were placed in cover glass slide chambers and were washed with ice-cold PBS. Cells were fixed with 3.7% formaldehyde diluted in DMEM for 30 min at room temperature. Following three washes with PBS, cells were blocked with a solution of 3% BSA and 0.2% TRITON X100 in PBS for 1 h. Cells at the basal level, as well as post DNA damage through etoposide, were stained for γ-H2AX, ATM, ATR, Chk1, and BRCA1 using a 1:200 primary antibody dilution, and incubated overnight at 4 °C on a rocker. Either secondary antibody AlexaFluor594 (594) (red) or AlexaFluor488 (AF488) (green) was added at a dilution of 1:500 for 1 h at room temperature. Cells were then stained with a dilution of 1:2000 DAPI and incubated in a dark room. Samples were imaged using ZEISS710 Confocal Laser Scanning Microscope (Carl Zeiss, Thornwood, NY, USA) confocal fluorescence microscopy at 40× magnification and analyzed using the SPOT imaging software (https://www.spotimaging.com/, accessed on 27 February 2025). For quantification of immunostaining, cells were counted after DMSO, Pon-A, ETOP-Pon-A, and ETOP-DMSO treatment, and an average number of cells containing concentrated foci were quantified and graphed. An average percent out of over 100 total cells were generated for each cell treatment and graphed.

### 2.5. Trypan Blue Assay

MEFs with indicated treatments were trypsinized and resuspended in PBS. Resuspended MEFs were mixed with 0.4% Trypan Blue solution (Sigma Aldrich, St. Louis, MO, USA) for 5 min at room temperature. The number of stained MEFs was quantified with a hemocytometer using a bright field microscope to indicate cell death.

### 2.6. Statistical Analysis

All calculations and graphs were performed and created using Microsoft Excel and IBM SPSS Statistics (9.0.2.0 (20)). All experiments were performed independently at least three times unless otherwise indicated. Student’s *t*-test was used to analyze statistical significance. *p*-values lower than 0.05 were considered statistically significant. Error bars are presented as ±1 standard error of the mean. *p*-Values are corrected for multiple testing using Benjamini Hochberg correction [27].

## 3. Results

### 3.1. Klf4^+/+^ MEFs Display Less DNA Damage After Etoposide Treatment

**Figure 1:**
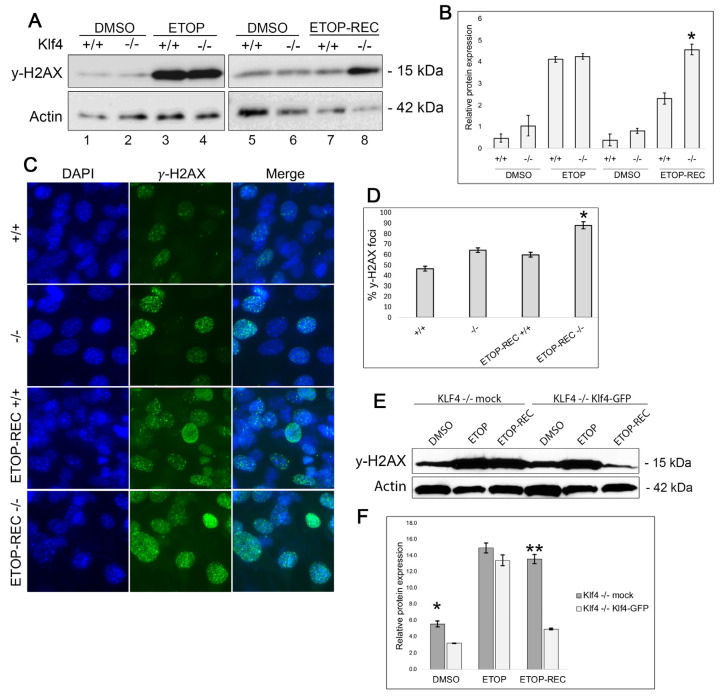
MEFs null for *Klf4* fail to effectively repair DNA damage following treatment with etoposide. (**A**) Western blot analysis indicates that *Klf4^−/−^* MEFs exhibit higher basal levels of DNA damage compared to *Klf4^+/+^* MEFs, as indicated by γ-H2AX (lanes 1 and 5 compared to lanes 2 and 6). Following treatment with etoposide for 5 h, both *Klf4^+/+^* and *Klf4^−/−^* showed high levels of γ-H2AX (lanes 3 and 4). However, *Klf4^−/−^* MEFs which were treated with etoposide for 5 h and had a chance to recover still acquired greater levels of DNA damage (lanes 5 and 7 compared with lanes 6 and 8). (**B**) Quantification of γ-H2AX Western blot normalized against β-actin. Error bars represent ±1 standard error (*n* = 3); * indicates *p* < 0.05. (**C**) MEFs were plated on chamber slides and either treated with 60 µM etoposide (ETOP) or DMSO as a control for 5 h and allowed to recover in fresh media overnight (ETOP-REC). Cells then were fixed in 3.7% formaldehyde and blocked with a blocking solution in PBS. Cells were incubated overnight with 1:200 γ-H2AX primary antibody, then stained with DAPI (blue) to stain the nucleus and AF488 (green). Images were taken using a fluorescence microscope at 40×. Scale bar for all panels: 50 μm. (**D**) Quantification of prominent γ-H2AX stain from ~100 cells per treatment: *Klf4^+/+^*—21%, *Klf4^−/−^*—30%, ETOP-REC *Klf4^+/+^*—29%, and ETOP-REC *Klf4^−/−^*—41.1%. Error bars represent ±1 standard error (*n* = 5). * indicates *p* < 0.05. (**E**) Proteins were extracted 24 h after transfection and either treated with 60 µM etoposide (ETOP) or DMSO as a control for 5 h and allowed to recover in fresh media overnight (ETOP-REC). γ-H2AX expression on *Klf4^−/−^* and *Klf4^−/−^* transfected with Klf4 were performed using Western blots. (**F**) Quantification of γ-H2AX Western blot normalized against β-actin. Error bars represent ±1 standard error (*n* = 3). * indicates *p* < 0.05; ** indicates *p* < 0.01.

We previously showed that *Klf4^+/+^* MEFs demonstrated lower levels of γ-H2AX, a biomarker for DNA damage, than *Klf4^−/−^* MEFs at the basal level [13]. To characterize the role of KLF4 in response to DNA damage we treated cells with etoposide, a topoisomerase poison, to induce DNA damage and then used Western blots for *Klf4^+/+^* and *Klf4^−/−^* MEFs before and after etoposide treatment (Figure 1A,B). It was found that *Klf4^+/+^* and *Klf4^−/−^* MEFs treated with etoposide for 5 h with no recovery showed similarly high levels of DNA damage (Figure 1A,B, Lanes 3 and 4). However, *Klf4^+/+^* MEFs treated for 5 h and allowed 24 h of recovery showed lower levels of γ-H2AX as compared to the *Klf4^−/−^* MEFs (Figure 1A,B, Lanes 7 and 8). These findings suggest that cells with functional Klf4 can recover more efficiently from DNA damage than cells null for *Klf4*.

In addition, we visualized the expression of γ-H2AX using immunofluorescence at the basal level and after 24 h of recovery from DNA damage (Figure 1C,D). Consistent with our previous publications, *Klf4^+/+^* MEFs show less γ-H2AX foci compared to *Klf4^−/−^* MEFs at the basal level [13]. In addition, we found that *Klf4^+/+^*-expressing cells treated with etoposide and allowed to recover showed lower levels of DNA damage (Figure 1D). Taken together, these data suggest Klf4 plays a role in maintaining genomic stability at the basal level and after the induction of DNA damage.

To further validate these findings, we reintroduced Klf4 expression in *Klf4^−/−^* MEFs by transfecting them with the Klf4 plasmid or mock control (Figure 1E). This was followed by Western blotting for γ-H2AX to determine relative DNA damage levels. Mock-transfected *Klf4*-null cells treated with etoposide showed similar γ-H2AX levels compared with no-recovery and 24 h-recovery groups. However, in comparison to the *Klf4^+/+^, Klf4^−/−^* MEFs that were transfected with the Klf4 plasmid and allowed to recover after etoposide treatment for 24 h exhibited significantly lower γ-H2AX levels in comparison to the no-recovery group treated with etoposide. Reintroducing Klf4 expression into cells deficient for Klf4 supports the role of Klf4 in DNA repair mechanisms and genomic stability (Figure 1F). Overall, our results show that in the absence of Klf4, there are higher levels of γ-H2AX in MEFs.

### 3.2. RKO Cells Expressing KLF4 Show Increased BRCA1 24 h After Etoposide-Induced DNA Damage

**Figure 2:**
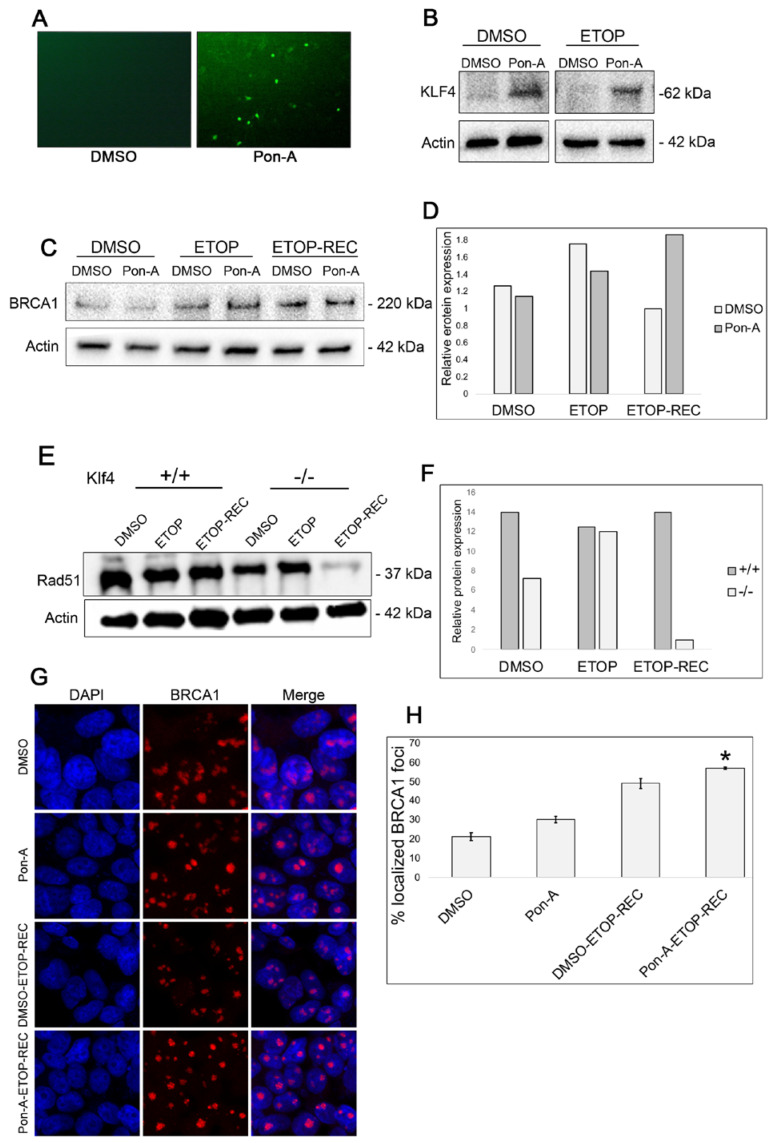
Klf4 expression leads to an increase in BRCA1 expression upon DNA damage. RKO cells were treated with 10 µM Pon-A to induce KLF4 expression and GFP fluoresce, or DMSO was used as a control. (**A**) Fluorescence images of cells treated with DMSO as a control or Pon-A show a GFP fluorescence signal indicating KLF4 is expressed. (**B**) Western blot confirming KLF4 expression using β-actin as a loading control for before and after DNA damage by etoposide. (**C**,**D**) RKO cells were treated with either 10 µM of Pon-A or DMSO as a control. Cells were then treated with either 60 µM of etoposide or DMSO as a control. (**C**) Western blot analysis was carried out to visualize the expression of BRCA1 using β-actin as a control at the basal level (DMSO), immediately after a 5 h treatment of 60 µM etoposide (ETOP), or treated and then washed with fresh DMEM for 24 h (ETOP-REC). (**D**) Quantification of Western blot relative to β-actin levels. (**E**) Western blot analysis was carried out to visualize the expression of Rad51 using β-actin as a control at the basal level (DMSO), immediately after a 5 h treatment of 60 µM etoposide (ETOP), or treated and then washed with fresh DMEM for 24 h (ETOP-REC). (**F**) Quantification of Western blot relative to β-actin levels. (**G**) Cells were fixed, blocked, and incubated with BRCA1 antibodies then stained with DAPI (blue) to stain the nucleus and AF594 (red). Images were taken using a confocal fluorescence microscope at 40×. (**H**) Quantification of the percentage of observed localized BRCA1 in the nucleus. A total of 300 cells per treatment were counted. The observed percent of BRCA1 concentrated foci is represented. DMSO showed 26% localized, Pon-A showed 29%, DMSO-ETOP-REC showed 49%, and Pon-A-ETOP-REC showed 57% localized BRCA1. Error bars represent ±1 standard error (*n* = 3). * indicates *p* < 0.05.

To investigate the role of KLF4 in DNA repair mechanisms, we used a stable cell line, RKO-EcR-KLF4 (RKO), that was previously transfected with a plasmid to induce KLF4 and GFP expression only when treated with Ponasterone-A (Pon-A) [9]. We observed GFP fluorescence in Pon-A-treated RKO cells compared with RKO cells treated with DMSO (Figure 2A). We then performed a Western blot for KLF4 to confirm that the GFP fluorescence was indicating KLF4 expression the RKO cells (Figure 2B).

Previous studies have shown BRCA1 is involved in DNA repair and genomic stability maintenance [28]. Additionally, γ-H2AX plays an important role in recruiting proteins such as BRCA1 to sites of double-strand breaks, further helping induce homologous recombination (HR) and DNA damage repair [29,30]. To test if Klf4 is associated with BRCA1 function through γ-H2AX, Western blots on RKO cells treated with Pon-A or DMSO were examined at the basal level, immediately after etoposide-induced DNA damage (ETOP), and 24 h post etoposide recovery (ETOP-REC) (Figure 2C,D). We found that at the basal level and immediately following etoposide DNA damage, there is an increase in BRCA1 levels in RKO cells not expressing KLF4 expression, compared to RKO cells expressing KLF4 (Figure 2D). This provides evidence for KLF4’s role in enhanced DDR. Interestingly, when the RKO cells were allowed time to recover, BRCA1 expression levels were elevated in KLF4-expressing RKO cells (Figure 2D). These results suggest that when RKO cells, expressing Klf4, are treated with etoposide and allowed to recover, KLF4 may somehow facilitate homologous recombination leading to localized BRCA1 at sites of DNA damage.

To determine a possible difference between *Klf4^+/+^* and *Klf4^−/−^* MEFs during the DNA damage response, a Western blot was conducted for Rad51, a protein involved in homologous recombination. The data are consistent with previously published findings that cells lacking *Klf4* show less expression of Rad51 (Figure 2E,F) [5]. In addition, when DNA damage was induced by etoposide with no recovery, cells lacking *Klf4* show lower expression of Rad51 compared with *Klf4* expressing cells. This result suggests that in the absence of *Klf4*, Rad51 may not be properly recruited to sites of DNA damage (Figure 2E).

To further understand the relationship between KLF4 and BRCA1, immunostaining was employed (Figure 2G). At the basal level, RKO cells not expressing KLF4 show approximately 26.5% concentrated BRCA1 foci (Figure 2H) as compared to RKO cells expressing KLF4, which show 29% concentrated BRCA1 foci (Figure 2H). We then performed an immunostaining on RKO cells treated with either DMSO or Pon-A. We found that cells not expressing Klf4 and treated with etoposide exhibited BRCA1 foci in 49% of the cells (Figure 2H). On the other hand, BRCA1 foci were displayed in 57% of RKO cells treated with etoposide and Pon-A (Figure 2H). Taken together, it appears KLF4 may not functionally enhance BRCA1 expression; however, it could lead to a clearer BRCA1 function at the post-translational level.

### 3.3. Genes Involved in DNA Repair Are Upregulated in Klf4 Expressing Cells

**Figure 3:**
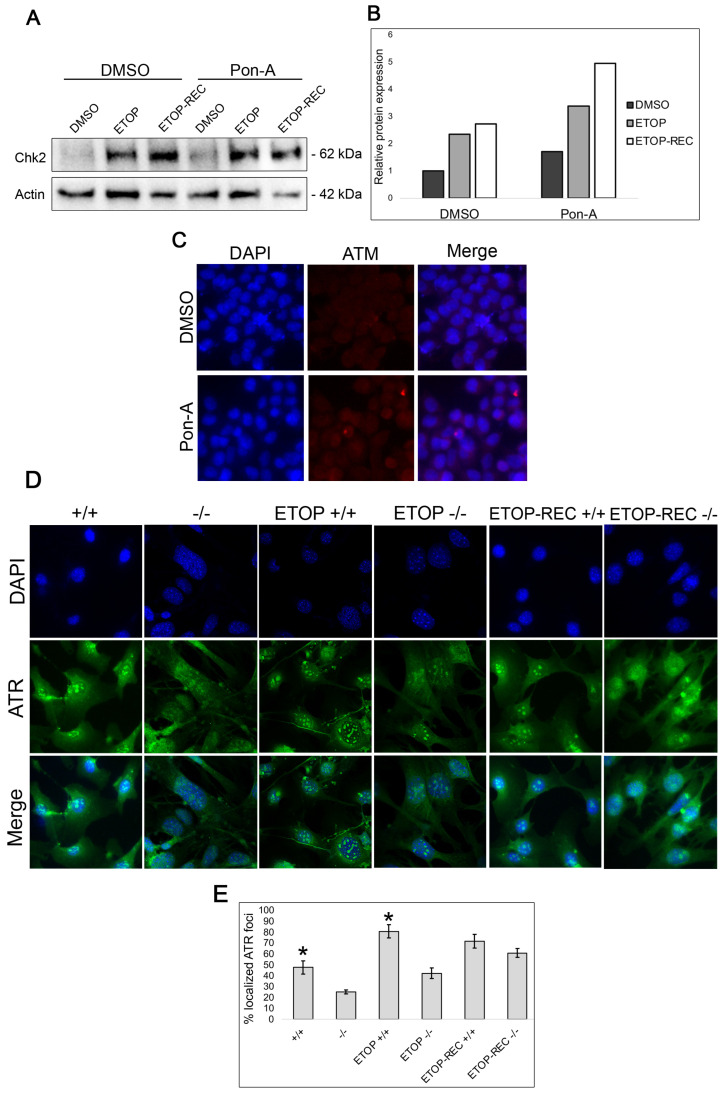
Chk2 levels are increased in RKO cells expressing KLF4 at the baseline and 24 h after etoposide treatment. RKO cells were treated with Pon-A or DMSO as a control. Cells were then treated with 60 µM of etoposide or DMSO as a control for 5 h and collected immediately (ETOP) or washed with fresh media and allowed to recover for 24 h (ETOP-REC). (**A**) Western blot analysis was carried out to visualize the expression of Chk2 using β-actin as a loading control. (**B**) Quantification of Chk2 Western blot normalized against β-actin. (**C**) Immunofluorescence stain on RKO cells treated with Pon-A or DMSO and tagged with ATM primary antibodies, and then stained with DAPI (blue) to stain the nucleus and AF594 (red) (*n* = 2). Images were taken at 40×. (**D**) Immunofluorescence stain on *Klf4^+/+^* and *Klf4^−/−^* MEFs were plated on chamber slides and treated with etoposide and visualized immediately after 5 h of treatment (ETOP) or after 24-hrecovery (ETOP-REC) tagged with ATR primary antibodies then stained with DAPI (blue) to stain the nucleus and AF488 (green). Images were taken at 40×. (**E**) Quantification of immunofluorescence for ATR. Error bars represent ±1 standard error (*n* = 3). * indicates *p* < 0.05.

To identify the relationship between KLF4 and DNA damage repair proteins within the ATM/ATR mediated DDR cascade, Western blots were performed for RKO cells expressing KLF4 upon Pon-A or DMSO treatment at the basal level, immediately following etoposide treatment, and after 24 h of recovery time post etoposide treatment (Figure 3A). These results suggest that at the basal level, there is an increase in Chk2 expression in RKO cells that express KLF4 compared to cells lacking functional KLF4. Furthermore, immediately after DNA damage induced by etoposide and after 24 h of recovery time, there is an increased Chk2 expression in Pon-A treated RKO cells expressing KLF4 compared to control RKO cells lacking *KLF4* expression (Figure 3B).

To further analyze the role of *Klf4* in DNA repair, immunostaining was performed for ATM, a kinase protein involved in DNA repair pathways, in RKO cells treated with or without Pon-A. We found no difference in ATM expression in the presence or absence of KLF4 at the basal level and after DNA damage (Figure 3C). The data indicated that *Klf4* does not regulate ATM expression. We then performed immunostaining for ATR in *Klf4^+/+^* and *Klf4^−/−^* MEFs to determine if the presence of *Klf4* affected ATR expression (Figure 3D). An increase in ATR was found at the basal level and immediately after etoposide treatment in *Klf4^+/+^* compared with *Klf4^−/−^* MEFs (Figure 3E). These results suggest that *Klf4* is involved in the ATR-mediated DNA damage response.

### 3.4. Chk1 Expression Is Higher at the Basal Level and Immediately After Etoposide DNA Damage in KLF4-Expressing Cells

**Figure 4:**
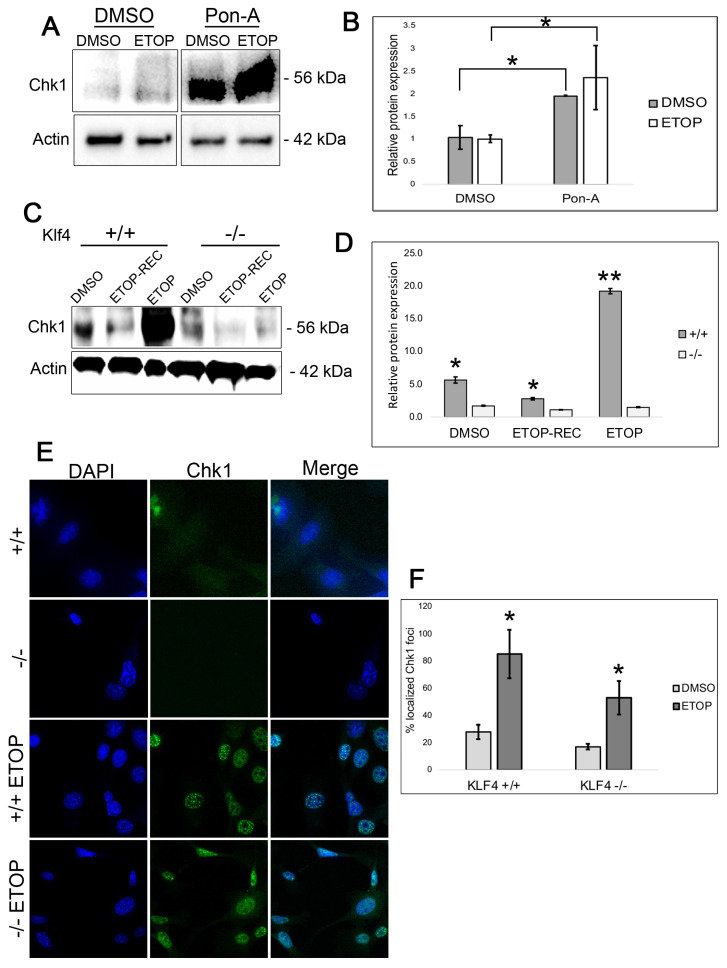
Cells expressing KLF4 exhibit greater Chk1 expression at the basal level and immediately following DNA damage. (**A**,**B**) RKO cells were treated with Pon-A or DMSO as a control. Cells were then treated with 60 μM of etoposide or DMSO as a control for 5 h and collected (ETOP). (**A**) Western blot analysis was carried out to visualize the expression of Chk1 using actin as a control. (**B**) Quantification of Chk1 Western blot normalized against β-actin. (**C**) Western blot representing Chk1 expression at the basal level, 48 h after etoposide treatment, or immediately after etoposide treatment in *Klf4^+/+^* and *Klf4^−/−^* MEFs. (**D**) Quantification of Chk1 Western blot normalized against β-actin. (**E**) *Klf4^+/+^* and *Klf4^−/−^* MEFs were treated with etoposide for 5 h and then treated immediately for immunofluorescence at 40×. MEFs stained with primary Chk1 antibody and stained with DAPI (blue) to stain the nucleus and AF488 (green). Images were taken using a fluorescence microscope at 40×. (**F**) Quantification of the percentage of cells showing Chk1 fluorescence. A total of 100 cells were counted per treatment and the average frequency of bright fluorescence is represented by a percentage. *Klf4^+/+^* showed 20% Chk1 and *Klf4^−/−^* showed 17% Chk1 fluorescence. Immediately after etoposide, *Klf4^+/+^* showed 58% Chk1 foci and *Klf4^−/−^* showed 38% Chk1 foci. Error bars represent ±1 standard error (*n* = 3). * indicates *p* < 0.05; ** indicates *p* < 0.01.

Chk1 is a DNA repair protein kinase and downstream target of ATR that plays an important role in cancer biology by mediating cell cycle regulation and the DNA damage response [30]. To investigate whether Chk1 expression is regulated by Klf4, we performed Western blots on RKO cells treated with Pon-A and cells treated with DMSO at both the basal level and immediately after etoposide treatment (Figure 4A,B). In the Pon-A-treated cells that induce Klf4, there was significantly greater Chk1 expression both at the basal level and immediately following etoposide treatment in comparison to the DMSO-treated cells lacking Klf4 expression (Figure 4A,B). Western blots examining *Klf4^+/+^* and *Klf4^−/−^* MEFs with no DNA damage and 48 h after etoposide treatment revealed that cells with functional Klf4 treated with etoposide showed higher levels of Chk1 (Figure 4C,D).

We then conducted immunostaining on *Klf4^+/+^* and *Klf4^−/−^* MEFs at the basal level and immediately after 5 h of etoposide treatment (Figure 4E). At the basal level, both the *Klf4^+/+^* and *Klf4^−/−^* display diffuse Chk1 fluorescence (Figure 4E). Upon quantification of these cells, the *Klf4^+/+^* MEFs showed a slightly higher frequency of Chk1 foci in the nucleus at 20% in the *Klf4^+/+^* compared to 17% in the *Klf4^−/−^* (Figure 4F). Additionally, immediately after etoposide treatment, there were higher numbers of Chk1 foci in the *Klf4^+/+^* at 58%, compared to 38% in the *Klf4^−/−^* MEFs (Figure 4F). This may indicate enhanced recruitment of Chk1 to sites of DNA damage in cells containing functional Klf4 [31]. Taken together, our results indicate a possible relationship between KLF4 and Chk1 to mediate DNA damage repair in cells both at the basal level and immediately following cellular stress.

### 3.5. Cells Wild Type for Klf4 Experience Less Cell Death than Cells Null for Klf4

**Figure 5:**
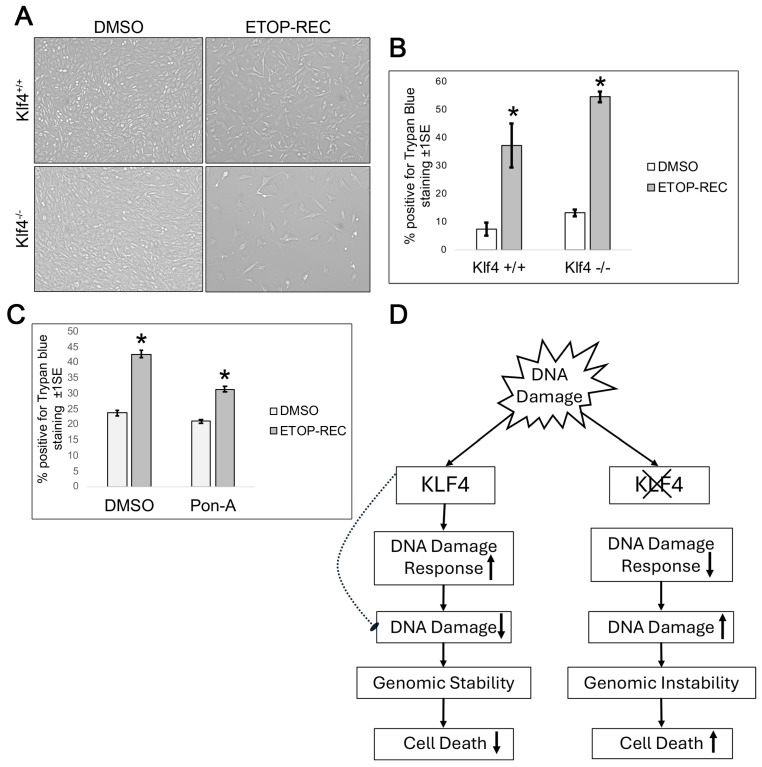
Cells expressing Klf4 experience less cell death after etoposide treatment. (**A**) Morphology of *Klf4^+/+^* and *Klf4^−/−^* MEFs treated with DMSO or 60 μM of etoposide for 5 h then washed with fresh media and allowed to recover for 24 h (ETOP-REC). (**B**) MEFs were collected after the respective treatments and subjected to a Trypan Blue assay. The percentage of dead cells, as indicated by Trypan Blue staining, was quantified using a hemocytometer under bright field microscopy. Error bars represent ±1 standard error (*n* = 5); * indicates *p* < 0.05. (**C**) RKO cells were collected after respective treatments and subjected to a Trypan Blue assay. The percentage of dead cells, as indicated by Trypan Blue staining, was quantified using a hemocytometer under bright field microscopy. Error bars represent ±1 standard error (*n* = 3). (**D**) Working model for KLF4 protein interaction following DNA damage. It is hypothesized that in the presence of KLF4, there is an increase in DNA damage response proteins involved in cell cycle arrest/repair, such as Chk1 and ATR. This results in less cell death and more genomic stability. On the other hand, cells lacking the *Klf4* gene exhibited more cell death due to less DNA repair and more genomic instability. Up-arrow depicts an increase and down-arrow depicts a decrease of the associated action.

To characterize the death response of DNA damage in *Klf4^+/+^* and *Klf4^−/−^* MEF cells, we performed a trypan blue assay (Figure 5A,B). After quantification, we found there was less cell death at the basal level and after etoposide treatment in the *Klf4^+/+^* in comparison to the *Klf4^−/−^* MEFs (Figure 5B). This indicates that cells containing functional Klf4 not only recover better but also exhibit higher cell survival rates post DNA damage.

To test the consistency of these findings across cell types, as well as to determine whether the induction of KLF4 alters cell viability, we ran a cell death assay in colorectal RKO cells. Pon-A-treated RKO cells with etoposide (ETOP-REC), showed less cell death compared to cells treated with DMSO (ETOP-REC) (Figure 5C). Consistent with our MEF wild type data (Figure 5A,B), a lower cell death was observed in RKO cells treated with Pon-A following etoposide treatment.

Overall, the data in this study further confirm that KLF4 is a tumor suppressor and proposes repair proteins that may be involved in the KLF4 regulatory pathway (Figure 5D). Our working model suggests that Klf4 upregulates known DNA repair proteins to decrease cellular damage and enhance cell vitality. On the other hand, cells null for *Klf4* showed lower expression of DNA repair genes, increasing genomic instability and cell death (Figure 5D).

## 4. Discussion

As hallmark of cancer, genomic instability can manifest as chromosomal aberrations, microsatellite instability, double-strand breaks, and frequent base-pair mutations [32]. It was previously reported that the Klf4 response to DNA damage is dependent on the severity of DNA damage [33]. Consistent with this finding, we also showed *Klf4*-null MEFs had high levels of genomic instability as compared to wildtype MEFs [12]. Moreover, we reported that restoration of Klf4-to-Klf4 MEFs reduces the extent of DNA damage and aneuploidy [10]. We have demonstrated that Klf4 reduces genomic instability by regulating antioxidant genes and reducing reactive oxygen species [2]. However, further research is necessary to determine whether Klf4 is directly involved in DNA repair mechanisms and DNA damage response (DDR).

Considering the previous studies that highlighted *Klf4*’s role in maintaining genomic stability, we investigated the mechanism of Klf4 in DNA repair using MEFs for the wild type (*Klf4^+/+^*) and null (*Klf4^−/^*^−^) variants. Further, using the DNA damage drug etoposide, a topoisomerase II poison, we further investigated the connection of Klf4 to the DNA damage response in RKO, human colorectal cancer cell lines. Overall, based on the Western blot analyses and immunostaining, our findings indicate that Klf4 is involved in DNA damage response pathways.

Specifically, our results indicate that KLF4 facilitates the localization of DDR factors, such as BRCA1 or Chk1, to the sites of DNA damage. It is possible that KLF4 may contribute to the formation of scaffolds or structures at damaged chromosomal sites, promoting the efficient recruitment of repair machinery. It has been reported that proteins like 53BP1 and BRCA1 form repair scaffolds, which enable the recruitment of downstream DDR factors [34]. KLF4 could act similarly by stabilizing chromatin or providing structural support to facilitate repair factor assembly at sites of DNA breaks. Additionally, KLF4 has been implicated in chromatin remodeling, which could support scaffold formation or localization of DDR factors [35].

We previously showed that MEFs lacking Klf4 exhibit higher levels of DNA damage [10,13]. Consistent with previous findings, we show here that MEFs lacking *Klf4* also display higher levels of DNA damage. We propose here that MEFs and RKO cells lacking Klf4 expression exhibit impaired DNA efficiency after etoposide treatment and subsequent recovery (Figure 1). Through Western blotting and immunostaining, we found that *Klf4^+/+^* MEFs exhibit lower γ-H2AX levels than *Klf4^−/−^* MEFs after etoposide treatment. This result indicates that cells containing functional Klf4 experience better recovery and higher levels of survival post DNA damage (Figure 1 and Figure 5). This further supports the results of Yoon et al., who found that KLF4 reduces chromosome abnormalities after introducing DNA damage [36]. Further, our current study showed that when Klf4 is reintroduced into cells lacking *Klf4*, γ-H2AX levels are decreased, indicating less DNA damage (Figure 1).

Next, we investigated whether KLF4 facilitates the localization of the tumor suppressor gene, BRCA1, a widely studied protein involved in checkpoints in response to DNA damage [37,38,39]. A study by Zhou et al. showed that KLF4 binds to the BRCA1 promoter to regulate its transcription [40]. Although we did not observe differences in the expression of BRCA between Pon-A-treated and DMSO-treated RKO cells, we were interested if KLF4 plays a role in the localization and stability of BRCA1 at the site of damage. To visualize whether KLF4 recruits BRCA1 to a DNA damage site, we conducted immunostaining for BRCA1. We found that RKO cells expressing KLF4 exhibited greater BRCA1 localization in the nucleus (Figure 2). This indicates that KLF4 is involved in recruiting the BRCA1 foci to specific sites of DNA damage. However, it is possible that KLF4 regulates BRCA1 at post-translational modifications, but this awaits further investigation.

Cells treated with etoposide, a topoisomerase II poison, are unable to unwind the double helix for DNA replication, promoting DNA double-strand breaks [25,41]. However, the study by Rossi et al. (2006) showed that when Chk1 is expressed, the lethality of etoposide is reduced due to its ability to minimize the replication damage caused by etoposide treatment [25]. Since etoposide poisons topoisomerase II, it was found to affect the redistribution of replication proteins involved in the ATR checkpoint [24,25]. Our findings display that cells deficient in *Klf4* exhibit low levels of Chk1 expression both at the basal level and immediately after etoposide treatment (Figure 4). This finding could support the greater levels of γ-H2AX, an indicator of DNA damage, observed in cells lacking functional KLF4. However, whether or not KLF4 influences Chk1 expression through direct regulation remains unknown.

Previous studies suggest an important relationship between Chk1 and BRCA1 after inducing DNA damage [42,43,44]. These studies note a relationship between the increase in Chk1 influencing homologous recombination as a mechanism for responding to DNA damage. Since etoposide causes damage through replication stress, Chk1 may be upregulated in response to alleviate the decreasing number of DNA double-strand breaks, to ensure cellular repair at the G2/M checkpoint. Further, it is possible that Chk1 compensates for the lack of homologous recombination and low γ-H2AX, which was similarly found in a recently published study [45]. This could explain what our results illustrated in Figure 2, where an increase in BRCA1 and γ-H2AX is demonstrated in RKO cells expressing KLF4 as compared with RKO cells not expressing KLF4 after 24 h of recovery. Moreover, there was an increase in Chk2 expression in the RKO cells expressing KLF4 compared to the cells not expressing KLF4 at both the basal level and after etoposide treatment (Figure 3). While there appears to be more Chk2 expression when KLF4 is present, this increase is not as drastic as the contrast in Chk1 expression between the cells expressing and lacking KLF4. This suggests that the DNA repair mechanism of KLF4 is more closely related to the ATR/Chk1 pathway. Chk2 has been found to operate with the ATM path and not the replication response of etoposide-induced stress [46]. Although our results do not show significant differences in ATM expression in cells with functional and non-functional KLF4, it is possible KLF4 could act in the ATM/CHK2 pathway (Figure 3A–C). However, our data did not eliminate the possibility that Klf4 could regulate Chk2 independently of ATM, perhaps at the transcriptional level. In addition, phosphorylation-based activation assays may further elucidate this discrepancy and provide insight into KLF4’s relationship with ATM and CHK2.

Our results show that both ATR and Chk1 are upregulated in the presence of KLF4 (Figure 3 and Figure 4). The findings of our study therefore suggest that Klf4 regulates ATR—a known Chk1 activator [30]. These two proteins are noted to play important roles in the replication arrest in the cell cycle, enabling cells to recover after DNA damage and cellular stress [47]. Klf4’s expected relationship with ATR/Chk1 agrees with the previous literature finding that KLF4 overexpression leads to an increase in cell cycle arrest at G1 [48,49,50]. However, further investigation will be required to determine whether KLF4 regulates ATR and CHK1 in a direct or indirect relationship. It is unknown if Klf4 solely upregulates ATR, a known regulator of Chk1, explaining the increase in Chk1 expression that is observed in cells that have functional Klf4. On the other hand, it is possible that KLF4 may regulate both ATR and Chk1 independent of one another. Therefore, future studies remain necessary to understand the possible downstream targets through which KLF4 is involved in DNA repair mechanisms.

Our data thus strongly argue that ATR/Chk1 may be a target of KLF4 that enhances cell cycle regulation, minimizes DNA damage, and promotes genomic stability [50,51]. Overall, our results indicate that KLF4 is involved in the DNA repair mechanism as a tumor suppressor gene. It will be important to continue to investigate the possibility of KLF4’s involvement in checkpoints to reduce the amount of damage during cellular replication. Like the transcription factor p53, KLF4 is known to upregulate the cyclin-dependent kinase inhibitor p21, halting the cell cycle at key checkpoints and enabling DNA repair to occur [52]. Moreover, we previously showed that MEFs null for *Klf4* are genetically unstable, as evidenced by the presence of DNA double-strand breaks [13]. Although p53 is upregulated in *Klf4*-null MEFs, the cells were still unable to repair the DNA damage [13]. This strongly suggests that Klf4 plays a role in repairing DNA damage independent of p53, but the exact mechanism of how KLF4 accomplishes this awaits further investigation. Our current findings indicate the involvement of KLF4 in DDR. Interestingly, Ghaleb et al., in 2016, hypothesized that KLF4 induces DNA repair in the colonic epithelium by reversing p53’s suppressive effects on homologous recombination (HR) and non-homologous end joining (NHEJ) [5]. The exact mechanism of how KLF4 promotes HR and NHEJ in the colonic epithelium remains unknown and warrants further investigation.

In 2015, Liu et al. found that MEFs null for *Klf4* exhibited downregulated antioxidant gene levels such as the gene GSTɑ4 [1]. This downregulation promotes the accumulation of reactive oxygen species (ROS), causing oxidative stress and increased rates of cellular damage [1]. Liu et al. proposed that the increased rates of oxidative stress induce premature cellular senescence as a defense mechanism in Klf4-null cells [1]. Cellular senescence ensures that damaged or mutated cells are not able to proceed through the cell cycle and undergo replication in mitosis. The presence of premature cellular senescence further supports our finding that cells null for Klf4 exhibit increased rates of genomic instability in comparison to cells containing functional Klf4, indicating Klf4’s potential role in DNA damage response pathways.

Furthermore, it may be of value to compare the effects of using etoposide and radiation as forms of inducing DNA damage in cells. Additional work is needed to further understand the role of KLF4 in cell cycle arrest, replication, and repair. Our study has further shown the vital role of Klf4 in enhancing genomic stability and has suggested novel DNA damage response pathways that Klf4 may be involved in. Specifically, Klf4 appears to be involved in the recruitment and prolonged stability of BRCA1 at sites of double-strand breaks in addition to activating the cell cycle regulator, CHK1, post DNA damage. It remains unknown if this regulation is direct or indirect, warranting further study including chromatin immunoprecipitation (ChIP) assays. Taken together, our findings strongly suggest that KLF4 is involved in DNA repair mechanisms to maintain genomic stability.

## Data Availability

To view the data collected for this study, please email Engda G. Hagos (ehagos@colgate.edu).

## References

[1] Liu C., DeRoo E.P., Stecyk C., Wolsey M., Szuchnicki M., Hagos E.G. (2015). Impaired autophagy in mouse embryonic fibroblasts null for Krüppel-like factor 4 promotes DNA damage and increases apoptosis upon serum starvation. Mol. Cancer.

[2] Liu C., La Rosa S., Hagos E.G. (2014). Oxidative DNA damage causes premature senescence in mouse embryonic fibroblasts deficient for Kruppel-like factor 4. Mol. Carcinog..

[3] Rosencrans W., Walsh Z., Houerbi N., Blum A., Belew A., Chernak B., Liu C., Trazo A., Olson A., Brauer P. (2020). Cells deficient for Krüppel-Like Factor 4 exhibit mitochondrial dysfunction and impaired mitophagy. Eur. J. Cell Biol..

[4] Li Z.-Y., Zhu Y.-X., Chen J.-R., Chang X., Xie Z.-Z. (2023). The role of KLF transcription factor in the regulation of cancer progression. Biomed. Pharmacother..

[5] Ghaleb A.M., Elkarim E.A., Bialkowska A.B., Yang V.W. (2016). KLF4 suppresses tumor formation in genetic and pharmacological mouse models of colonic tumorigenesis. Mol. Cancer Res..

[6] Yang V.W., Liu Y., Kim J., Shroyer K.R., Bialkowska A.B. (2019). Increased genetic instability and accelerated progression of colitis-associated colorectal cancer through intestinal epithelium-specific deletion of Klf4. Mol. Cancer Res..

[7] Zhai F., Cao C., Zhang L., Zhang J. (2017). miR-543 promotes colorectal cancer proliferation and metastasis by targeting Klf4. Oncotarget.

[8] He Z., He J., Xie K. (2023). KLF4 transcription factor in tumorigenesis. Cell Death Discov..

[9] Dang D.T., Chen X., Feng J., Torbenson M., Dang L.H., Yang V.W. (2003). Overexpression of Krüppel-like factor 4 in the human colon cancer cell line RKO leads to reduced tumorigenicity. Oncogene.

[10] El-Karim E.A., Hagos E.G., Ghaleb A.M., Yu B., Yang V.W. (2013). Krüppel-like factor 4 regulates genetic stability in mouse embryonic fibroblasts. Mol. Cancer.

[11] Yoon H.S., Chen X., Yang V.W. (2003). Krüppel-like factor 4 mediates p53-dependent G1/S cell cycle arrest in response to DNA damage. J. Biol. Chem..

[12] Zhao W., Hisamuddin I.M., Nandan M.O., Babbin B.A., Lamb N.E., Yang V.W. (2004). Identification of Krüppel-like factor 4 as a potential tumor suppressor gene in colorectal cancer. Oncogene.

[13] Hagos E.G., Ghaleb A.M., Dalton W.B., Bialkowska A.B., Yang V.W. (2009). Mouse embryonic fibroblasts null for the Krüppel-like factor 4 gene are genetically unstable. Oncogene.

[14] Mah L.J., El-Osta A., Karagiannis T.C. (2010). gammaH2AX: A sensitive molecular marker of DNA damage and repair. Leukemia.

[15] Thielhelm T.P., Goncalves S., Welford S., Mellon E.A., Bracho O., Estivill M., Brown C., Morcos J., Ivan M.E., Telischi F. (2021). Primary vestibular schwannoma cells activate P21 and RAD51-associated DNA repair following radiation-induced DNA damage. Otol. Neurotol..

[16] Fan Z., Luo H., Zhou J., Wang F., Zhang W., Wang J., Li S., Lai Q., Xu Y., Wang G. (2020). Checkpoint kinase-1 inhibition and etoposide exhibit a strong synergistic anticancer effect on chronic myeloid leukemia cell line K562 by impairing homologous recombination DNA damage repair. Oncol. Rep..

[17] Bartek J., Lukas J. (2003). Chk1 and CHK2 kinases in checkpoint control and cancer. Cancer Cell.

[18] Marín B.G., Calderón-Segura M.E., Sekelsky J. (2023). ATM/CHK2 and ATR/Chk1 pathways respond to DNA damage induced by Movento^®^ 240SC and Envidor^®^ 240SC keto-enol insecticides in the germarium of Drosophila melanogaster. Toxics.

[19] Reinhardt H.C., Yaffe M.B. (2009). Kinases that control the cell cycle in response to DNA damage: Chk1, CHK2, and MK2. Curr. Opin. Cell Biol..

[20] Zuco V., Benedetti V., Zunino F. (2010). ATM- and ATR-mediated response to DNA damage induced by a novel camptothecin, ST1968. Cancer Lett..

[21] Wu X., Xu S., Wang P., Wang Z.-Q., Chen H., Xu X., Peng B. (2022). ASPM promotes ATR-CHK1 activation and stabilizes stalled replication forks in response to replication stress. Proc. Natl. Acad. Sci. USA.

[22] Treszezamsky A.D., Kachnic L.A., Feng Z., Zhang J., Tokadjian C., Powell S.N. (2007). BRCA1- and BRCA2-deficient cells are sensitive to etoposide-induced DNA double-strand breaks via topoisomerase II. Cancer Res..

[23] Menendez D., Anand J.R., Murphy C.C., Bell W.J., Fu J., Slepushkina N., Buehler E., Martin S.E., Lal-Nag M., Nitiss J.L. (2022). Etoposide-induced DNA damage is increased in p53 mutants: Identification of ATR and other genes that influence effects of p53 mutations on TOP2-induced cytotoxicity. Oncotarget.

[24] Durinikova E., Reilly N.M., Buzo K., Mariella E., Chilà R., Lorenzato A., Dias J.M.L., Grasso G., Pisati F., Lamba S. (2022). Targeting the DNA Damage Response Pathways and Replication Stress in Colorectal Cancer. Clin. Cancer Res..

[25] Rossi R., Lidonnici M.R., Soza S., Biamonti G., Montecucco A. (2006). The dispersal of replication proteins after etoposide treatment requires the cooperation of Nbs1 with the ataxia telangiectasia Rad3-related/Rhk1 pathway. Cancer Res..

[26] Ponasterone A—Insect Steroid Hormones and Gene Switching. https://agscientific.com/blog/ponasterone-a-gene-switching.html.

[27] Noble W.S. (2009). How does multiple testing correction work?. Nat. Biotechnol..

[28] Savage K.I., Gorski J.J., Barros E.M., Irwin G.W., Manti L., Powell A.J., Pellagatti A., Lukashchuk N., McCance D.J., McCluggage W.G. (2014). Identification of a BRCA1-mRNA splicing complex required for efficient DNA repair and maintenance of genomic stability. Mol. Cell.

[29] Choi E.-H., Yoon S., Hahn Y., Kim K.P. (2017). Cellular dynamics of Rad51 and Rad54 in response to postreplicative stress and DNA damage in HeLa cells. Mol. Cells.

[30] Scully R., Chen J., Plug A., Xiao Y., Weaver D., Feunteun J., Ashley T., Livingston D.M. (1997). Association of BRCA1 with RAD51 in mitotic and meiotic cells. Cell.

[31] Zhang Y., Hunter T. (2014). Roles of Chk1 in cell biology and cancer therapy. Int. J. Cancer.

[32] Gorgoulis V.G., Halazonetis T.D., Negrini S. (2010). Genomic instability—An evolving hallmark of cancer. Nat. Rev. Mol. Cell Biol..

[33] Zhou Q., Hong Y., Zhan Q. (2009). Role for Kruppel-like factor 4 in determining the outcome of p53 response to DNA damage. Cancer Res..

[34] Panier S., Boulton S.J. (2014). Double-strand break repair: 53BP1 comes into focus. Nat. Rev. Mol. Cell Biol..

[35] Zhu W., Li X., Zhang Y., Tang F., Xu W. (2024). KLF4 facilitates chromatin accessibility remodeling in porcine early embryos. Sci. China Life Sci..

[36] Yoon H.S., Ghaleb A.M., Nandan M.O., Hisamuddin I.M., Dalton W.B., Yang V.W. (2005). Krüppel-like factor 4 prevents centrosome amplification following γ-irradiation-induced DNA damage. Oncogene.

[37] Deng C.-X. (2006). BRCA1: Cell cycle checkpoint, genetic instability, DNA damage response and cancer evolution. Nucleic Acids Res..

[38] Stok C., Kok Y.P., van den Tempel N., van Vugt M.A.T.M. (2021). Shaping the BRCAness mutational landscape by alternative double-strand break repair, replication stress and mitotic aberrancies. Nucleic Acids Res..

[39] Yoshida K., Miki Y. (2004). Role of BRCA1 and BRCA2 as regulators of DNA repair, transcription, and cell cycle in response to DNA damage. Cancer Sci..

[40] Zhou Z., Huang F., Shrivastava I., Zhu R., Luo A., Hottiger M., Bahar I., Liu Z., Cristofanilli M., Wan Y. (2020). New insight into the significance of KLF4 PARylation in genome stability, carcinogenesis, and therapy. EMBO Mol. Med..

[41] Montecucco A., Zanetta F., Biamonti G. (2015). Molecular mechanisms of etoposide. Excli. J..

[42] Narayanaswamy P.B., Tkachuk S., Haller H., Dumler I., Kiyan Y. (2016). Chk1 and RAD51 activation after DNA damage is regulated via urokinase receptor/TLR4 signaling. Cell Death Dis..

[43] Yao Q., Weigel B., Kersey J. (2007). Synergism between etoposide and 17-AAG in leukemia cells: Critical roles for Hsp90, FLT3, topoisomerase II, Chk1, and RAD51. Clin. Cancer Res..

[44] Yarden R.I., Pardo-Reoyo S., Sgagias M., Cowan K.H., Brody L.C. (2002). BRCA1 regulates the G2/M checkpoint by activating Chk1 kinase upon DNA damage. Nat. Genet..

[45] Meyer F., Becker S., Classen S., Parplys A.C., Mansour W.Y., Riepen B., Timm S., Ruebe C., Jasin M., Wikman H. (2020). Prevention of DNA replication stress by Chk1 leads to chemoresistance despite a DNA repair defect in homologous recombination in breast cancer. Cells.

[46] Théard D., Coisy M., Ducommun B., Concannon P., Darbon J.-M. (2001). Etoposide and Adriamycin but not Genistein can activate the checkpoint kinase Chk2 independently of ATM/ATR. Biochem. Biophys. Res. Commun..

[47] Smith J., Mun Tho L., Xu N., Gillespie D.A. (2010). The ATM–Chk2 and ATR–Chk1 pathways in DNA damage signaling and cancer. Adv. Cancer Res..

[48] Choi H., Roh J. (2018). Role of KLF4 in the regulation of apoptosis and cell cycle in rat granulosa cells during the periovulatory period. Int. J. Mol. Sci..

[49] Chen X., Johns D.C., Geiman D.E., Marban E., Dang D.T., Hamlin G., Sun R., Yang V.W. (2001). Krüppel-like factor 4 (gut-enriched krüppel-like factor) inhibits cell proliferation by blocking G1/S progression of the cell cycle. J. Biol. Chem..

[50] Li S., Wang L., Wang Y., Zhang C., Hong Z., Han Z. (2022). The synthetic lethality of targeting cell cycle checkpoints and PARPs in cancer treatment. J. Hematol. Oncol..

[51] Weber A.M., Ryan A.J. (2015). ATM and ATR as therapeutic targets in cancer. Pharmacol. Ther..

[52] Evans P.M., Liu C. (2008). Roles of Krüppel-like factor 4 in normal homeostasis, cancer and stem cells. Acta Biochim. Biophys. Sin..

